# Proteomics as a new tool to study fingermark ageing in forensics

**DOI:** 10.1038/s41598-018-34791-z

**Published:** 2018-11-06

**Authors:** Stijn Oonk, Tom Schuurmans, Martin Pabst, Louis C. P. M. de Smet, Marcel de Puit

**Affiliations:** 1 Netherlands Forensic Institute, Digital Technology and Biometrics, Laan van Ypenburg 6, 2497 GB Den Haag, Netherlands; 2 Delft University of Technology, Faculty of Applied Sciences, Department of Chemical Engineering, Van der Maasweg 9, 2629 HZ Delft, The Netherlands; 3 Delft University of Technology, Faculty of Applied Sciences, Department of Biotechnology, Van der Maasweg 9, 2629 HZ Delft, The Netherlands; 4 Wageningen University & Research, Laboratory of Organic Chemistry, Stippeneng 4, 6708 WE Wageningen, The Netherlands

## Abstract

Fingermarks are trace evidence of great forensic importance, and their omnipresence makes them pivotal in crime investigation. Police and law enforcement authorities have exploited fingermarks primarily for personal identification, but crucial knowledge on when fingermarks were deposited is often lacking, thereby hindering crime reconstruction. Biomolecular constituents of fingermark residue, such as amino acids, lipids and proteins, may provide excellent means for fingermark age determination, however robust methodologies or detailed knowledge on molecular mechanisms in time are currently not available. Here, we address fingermark age assessment by: (i) drafting a first protein map of fingermark residue, (ii) differential studies of fresh and aged fingermarks and (iii), to mimic real-world scenarios, estimating the effects of donor contact with bodily fluids on the identification of potential age biomarkers. Using a high-resolution mass spectrometry-based proteomics approach, we drafted a characteristic fingermark proteome, of which five proteins were identified as promising candidates for fingermark age estimation. This study additionally demonstrates successful identification of both endogenous and contaminant proteins from donors that have been in contact with various bodily fluids. In summary, we introduce state-of-the-art proteomics as a sensitive tool to monitor fingermark aging on the protein level with sufficient selectivity to differentiate potential age markers from body fluid contaminants.

## Introduction

Fingermarks are frequently encountered at crime scenes and on related items, and naturally protected against fast degradation due to the abundance of recalcitrant substances, such as fatty acids and constituents of sebum^1–3^. While representing a major and valuable source for donor identification through latent fingerprint analysis, fingermarks are also rich in molecules that can be used to acquire toxicological and biological donor profiling information. In addition, exogenous compounds in fingermarks have shown applicability for crime reconstruction and to assess offender and victim activities before, during and after a criminal act. Many of such applications have been reviewed by van Dam *et al*.^4^, and Huynh and Halamek^5^. A major limitation in these respects is the lack of temporal information, which makes it difficult to establish forensic timelines. At present, precise assessment of the age of a fingermark is not possible.

Although fingermark residue is recognized as an important source of tell-tale (bio)molecules, it is not yet routinely used for forensic profiling in casework or in court. Nonetheless, recent research efforts have now placed illicit substances (and their metabolites), lipids, amino acids, DNA, RNA and proteins high on the forensic research agenda^6–13^. Fingermarks likely contain low amounts of endogenous proteins, compared to abundantly present lipids, salts and free amino acids^3^, and have only attracted limited attention from a proteomics point of view. In contrast, contaminant proteins on fingers have been proved to be highly relevant in studying donor contact with bodily fluids, such as blood^8,10^ and vaginal fluid^14^ by means of mass spectrometry (MS) analysis. From previous studies on fingermarks and epidermal skin layers it can be deduced that fingermark residue holds a wide variety of slowly degradable keratins alongside more typical proteins originating from sweat^15–20^. Upon deposition on a substrate, the protein composition of fingermarks is likely to change, whereas rates of degradation as well as alterations originating from e.g. oxidation, deamidation or alkylation processes will vary, depending notably on environmental conditions and time. This makes these biomolecules good targets to assess crucial yet presently unavailable temporal information for crime investigation. Fluorescence-based screening of proteinaceous material in parallel with lipids has recently been introduced as a high-throughput, non-contact methodology to assess the age of fingermarks from forensic contexts^21^. Despite the successful assessment of potential spectral regions of interest and a preliminary age-estimation model, this study, however, did not focus on compound identification nor on specific degradation/expression pathways. Furthermore, studies that have attempted to identify endogenous proteins from fingermarks were restricted either to indirect methods (SDS-PAGE and Westernblot analyses^16^) or peptide mass fingerprinting combined with putative protein identification from the literature^8,9^. Hence, in the strictest sense, no sequence-based proteomics studies have been conducted solely on fingermark residue until now. Therefore, a first proteomic map of fingermark development in time would not only provide further information on the chemical composition of fingermark residue, but likely would also reveal ageing pathways and related biomarkers.

**Figure 1:**
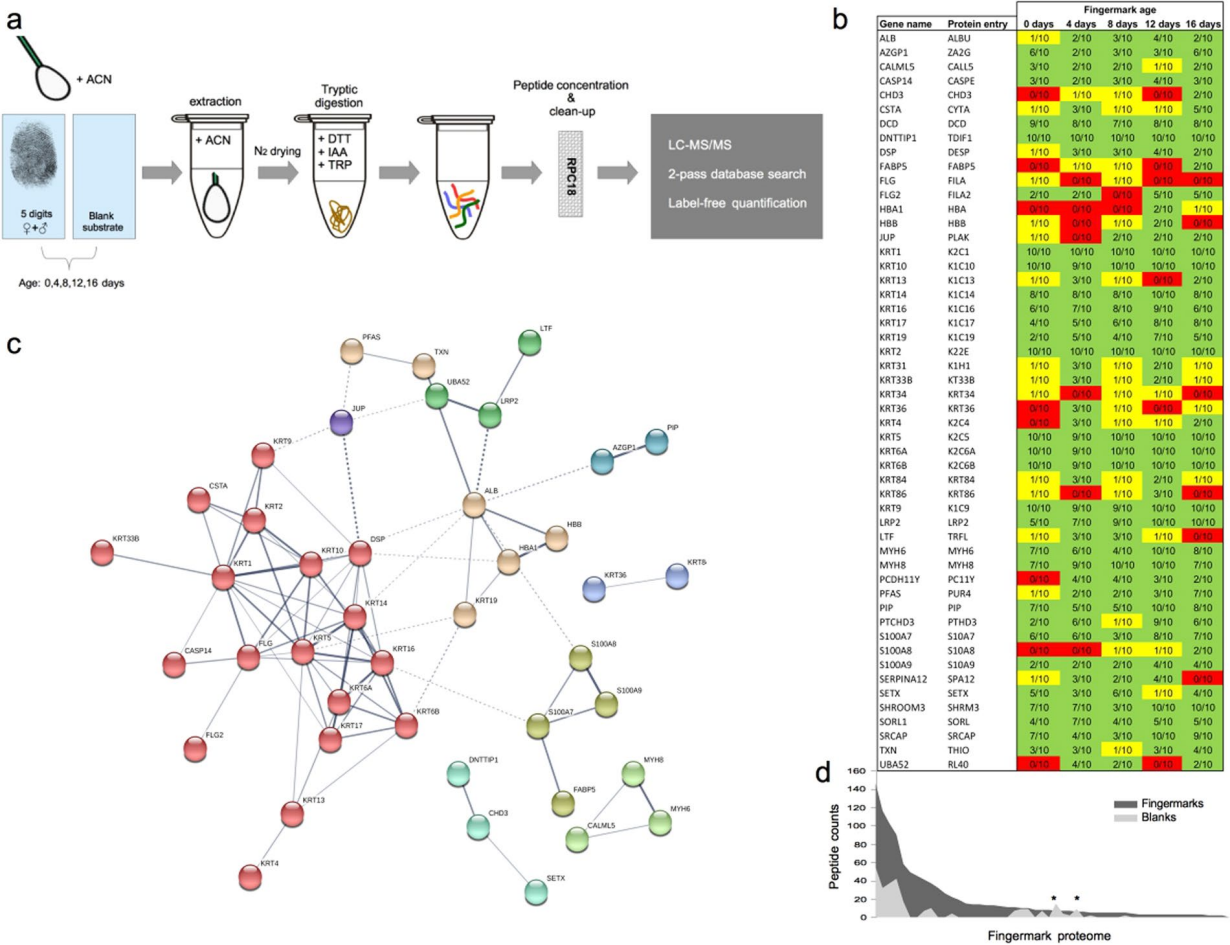
Overview of analytical approach and the identified fingermark proteome. (**a**) Overview of the analytical approach. (**b**) heatmap of all identified proteins; red (protein not identified in any sample pool), yellow (protein identified in 1 out of 10 fingermarks in particular sample pool) and green (protein identified in ≥2 out of 10 fingermarks in sample pools). Ratios (n/10) represent the number of fingermark samples in which a protein was identified for each sample pool (n = 10 samples). (**c**) Functional protein-protein associations of identified fingermark proteins and Markov clustering (inflation parameter = 2) obtained through the STRING Network. The proteins (nodes) are represented by their gene names and functional associations are indicated by the grey lines. Their level of confidence is given by the thickness of the lines, ranging from 0.150 (dotted lines) and 0.900 (dark grey). Protein clusters are indicated by same-colour nodes; red (cluster 1), brown (cluster 2), olive green (cluster 3), blue (cluster 4), mint (cluster 5), light purple (cluster 6), light green (cluster 7), dark green (cluster 8) and dark purple (cluster 9). (**d**) Coverage of the obtained fingermark proteome (x-axis, ordered high-to-low peptide counts from left to right) in combined fresh and aged procedural blanks (n = 10, light grey area) and thumb marks from female donors (n = 10, dark grey area). * indicates proteins, DESP and SETX respectively.

In this study, we developed a fingermark sampling and protein extraction protocol easily adaptable to forensic settings. A bottom-up proteomics approach (Fig. 1a) was then applied to confidently identify protein traces and assess the fingermark proteome dynamic changes at different stages of ageing (hereafter referred to as ‘ageing study’). This approach was also applied to contaminated fingermarks to examine fingermark ageing in light of realistic forensic scenarios and detect donor contact with bodily fluids (hereafter referred to as ‘donor contact study’). Unrestrictive searches of these proteomics data were also performed to assess protein damage and modification during ageing.

Here, a fingermark proteome of about 50 proteins was recorded, whereof a small fraction of keratin proteins and the antimicrobial peptide dermcidin (DCD) exhibited distinct responses during ageing. Ageing effects included an early increase in the frequency of observed amino acid residue modifications as well as protein degradation processes, decreasing the number of observable peptides. In addition, we demonstrate that most of the here described endogenous fingermark proteins can even be measured after contact with bodily fluids. In all, we drafted a first fingermark protein map including candidates that may serve as novel markers for accessing fingermark aging in forensics.

## Results

The relatively low protein content of fingermark residue as discerned from the literature was confirmed by a separate study using a bicinchoninic acid (BCA) assay and nanodrop spectrophotometric analysis for protein quantification; Protein yields were inferred to range from about 0.2 to 51.0 μg per fingermark (see Supplementary Material). For the ageing study, protein searches against a focussed fingermark protein database resulted in the identification of 52 proteins across all ageing pools of which 31 were redundant between these sample pools. Unique proteins were only identified in fresh fingermark residue (n = 2), and in marks aged for 8 days (n = 1) and 12 days (n = 1) (Supplementary Fig. S1). In the donor contact study, positive identifications of 77 (saliva), 36 (urine) and 53 (vaginal fluid) proteins could be obtained from two-pass Andromeda searches against the protein database followed by a focussed database search.

### Proteins identified from fingermarks

The majority of the proteins reported in this work (Fig. 1b and Supplementary Table S7) have, to the best of our knowledge, never been previously identified through protein analysis from fingermarks. Only keratin, type II cytoskeletal 1 (K2C1) and Keratin, type I cytoskeletal 10 (K1C10), Serum albumin (ALBU) and DCD have been isolated from fingermarks in other studies using Westernblot analysis and/or immunolabeling^15,16^. In contrast to these studies, Cathepsin-D (CATD) could not be identified from our data. The recorded proteomic profile is in line with that observed from the skin surface^18,19^, with cytokeratins as dominant protein species. These components make up approximately 35% of the fingermark proteome, whilst the remainder is accounted for by an array of antibiotic proteins as well as secreted blood proteins^22–26^. Gene ontology (GO) analysis (Supplementary Fig. S2) revealed that most proteins reported here are intermediate filament components, whilst another fraction has an extracellular origin. Similar grouping is displayed by the functional and biological process annotation; structural proteins evidently involved in skin development and keratinocyte differentiation, which prevail in fingermark residue, and homeostatic proteins that form a smaller subset. Protein interactions and functional relationships were obtained through the STRING database and Markov clustering^27^ (Fig. 1c). For the 52 identified proteins, approximately 80% (n = 43 proteins) could be associated with nine clusters. These clusters show the expression of two prominent protein pathways i.e.; keratinocyte proliferation (clusters 1, 5, 6 and 9) and the secretion of proteins likely originating from eccrine sweat^28^ (clusters 2,3,4). Cluster 1 consists primarily of keratins and includes a more or less separate group of non-keratins, including Filaggrin and Filaggrin-2 (FILA, FILA2), Caspase-14 (CASPE), Cystatin-A (CYTA) and Desmoplakin (DESP). The second most dominant clusters are represented by serum proteins; ALBU and Hemoglobin subunit beta (HBB), as well as Keratin type I cytoskeletal 19 (K1C19) and redox associated proteins Thioredoxin (THIO) and Phosphoribosylformylglycinamide synthase (PUR4) (cluster 2), and proteins likely representing sweat induced species associated with (non)immune defence against pathogens (cluster 3: proteins S100-A7, 8 and 9 (S10A7, S10A8, S10A9)) and Fatty acid-binding protein (FABP5) and cluster 4: Zinc-alpha-2-glycoprotein (ZA2G) and Prolactin-inducible protein (PIP)). Other groupings in our data are associated with more specific processes i.e.; chromatin interactions (cluster 5: Chromodomain-helicase-DNA-binding protein 3 (CHD3), Deoxynucleotidyltransferase terminal-interacting protein 1 (TDIF1) and Probable helicase senataxin (SETX) and hair formation (cluster 6: Keratin, type I cuticular Ha6 (KRT36) and Keratin, type II, cuticular Hb4 (KRT84)). Other linked proteins in the here observed fingermark proteome were primarily based on interactions between putative homologs found in species other than humans (clusters 7, 8). Lastly, a single-member cluster containing only Junction plakoglobin (PLAK), a protein associated with keratinocyte adhesion, was found to link clusters 1, 8 and 9. The clustering of our data is largely in agreement with origin of fingermark residue i.e.; material shed from skin and sweat, and this further supports that the proteome reported is a genuine molecular representation of fingermarks. As previously observed and confirmed by this work, high abundance proteins in fingermarks are primarily keratins. Specifically, keratins 1, 2, 5, 6, 9, 10, 14 and 17 were observed in almost all individual fingermarks with up to 57% sequence coverage. Other proteins (including some other keratins) are typically represented only by some of the fingermarks (see Fig. 1b).

The procedural blanks (n = 10), used in this work to monitor background contamination, displayed low coverage of the here observed fingermark proteome (Fig. 1d). This might be due to carry-over effects and/or common contamination e.g. from dust. For two proteins (DESP, SETX), peptide counts were however observed higher than the selected fingermark samples (n = 10). As these proteins are generally not regarded as common contaminants in MS-based proteomics experiments, it is likely that this background originates from carry-over between sample analysis runs.

### Ageing study

**Figure 2:**
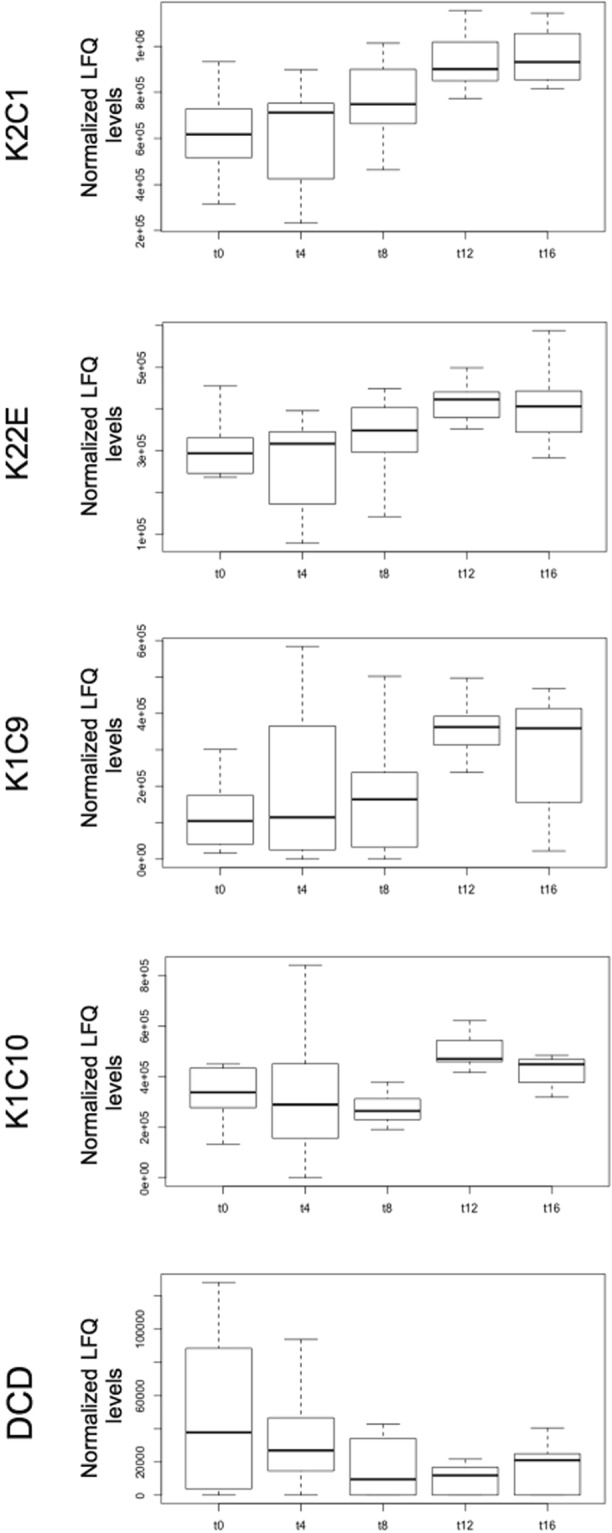
Normalized protein abundance levels (LFQ) for potential ageing markers. Boxplots showing the distribution of potential ageing markers K2C1, K22E, K1C9, K1C10 and DCD over time. Fingermark ages are indicated by t (0, 4, 8, 12 and 16) and are given in days. For these proteins ANCOVA showed significant ageing effects, whilst non-significant effects were for donor sex and type of digit (see Supplementary Table S1). Specific differences between fingermark ages for each protein were assessed by pairwise t-tests (see Supplementary Table S2).

For the 31 proteins that were identified in all sample pools, we performed ANCOVA using fingermark age, donor sex and finger type as outcome variables. Next, the normalized abundances of these proteins were manually evaluated for changes with increased age. Combined ageing effects and clear temporal expression trends were found for K2C1, Keratin, type II cytoskeletal 2 epidermal (K22E), Keratin, type I cytoskeletal 9 (K1C9), K1C10 and DCD. Here, effects of ageing were all significant, whereas donor sex or finger type effects were non-significant (see Supplementary Table S1) and hence we selected these proteins as potential age biomarkers. As shown in Fig. 2, all keratin proteins reveal a tipping-point threshold at 8 days of ageing; normalized abundance levels for these proteins were found to increase after this time period. Reversely, a decreasing trend was observed for DCD. Although ageing effects for the keratin proteins were, on the whole, significant as obtained from ANCOVA (Supplementary Table S1), stringent ad-hoc assessment of the ageing trends through Bonferroni corrected t-tests revealed that pairwise LFQ differences were significant for only a few time-points. Moreover, for DCD no significant changes in abundance were evident with fingermark age (Supplementary Table S2). Other protein members of the fingermark proteome presented here did not show particular trends nor significant effect of ageing.

### Protein modifications

Double-blind searches were performed using MODa software and used to detect protein modifications commonly associated with protein damage and ageing. Apart from single, double and triple alkylation (mass shifts: +57, +114 and +171 Da), disulphide reduction (mass shift: +2 Da), ammonia loss (−17 Da) and potassium adducts (+39 Da) likely coming from sample preparation or electrospray ionization, top 10 abundant mass shifts common to K2C1, K22E, K1C9, K1C10, and DCD were found to be at: −16, −48, +1, +16, +30, +52, +53, +56, +73, +104 Da. These mass shifts were interpreted using the unimod database (www.unimod.org) as deoxydation, dethiomethylation, deamidation, oxidation or hydroxylation, hydroxymethylation, cys-arg substitution, diethylation and carboxyethylation. These modifications are however not equally distributed amongst the ageing sets and we were unable to assign mass shifts at +52 and +104 Da to potential modification events.

**Figure 3:**
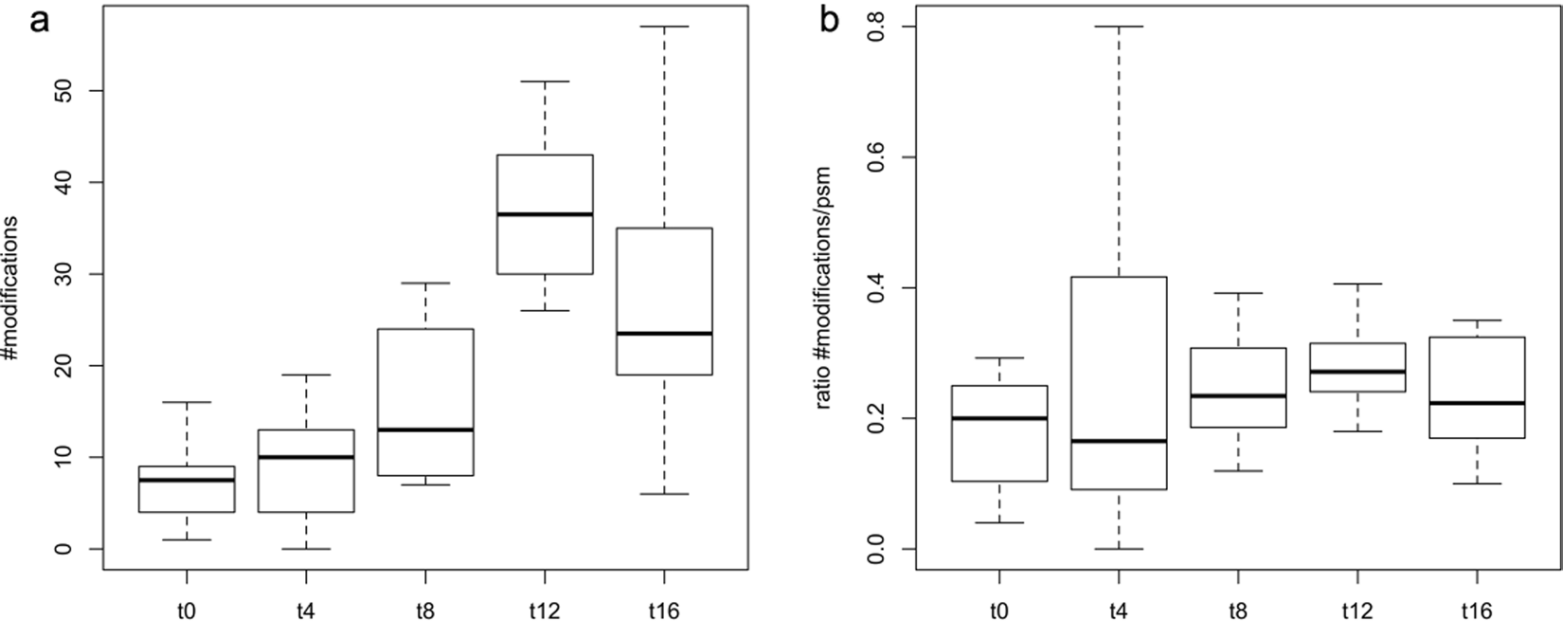
Age-biomarker modifications in fresh and aged fingermarks. Distribution of the observed modifications for potential ageing markers K2C1, K22E, K1C9, K1C10 and DCD over time, measured as (**a**) total numbers of mass shifts and (**b**) fraction of total peptide-spectrum-matches (psm). Fingermark ages are indicated by t (0, 4, 8, 12 and 16) and are given in days on the x-axis. Note that usual sample preparation and MS analysis induced mass shifts or adducts are not taken into account here.

**Figure 4:**
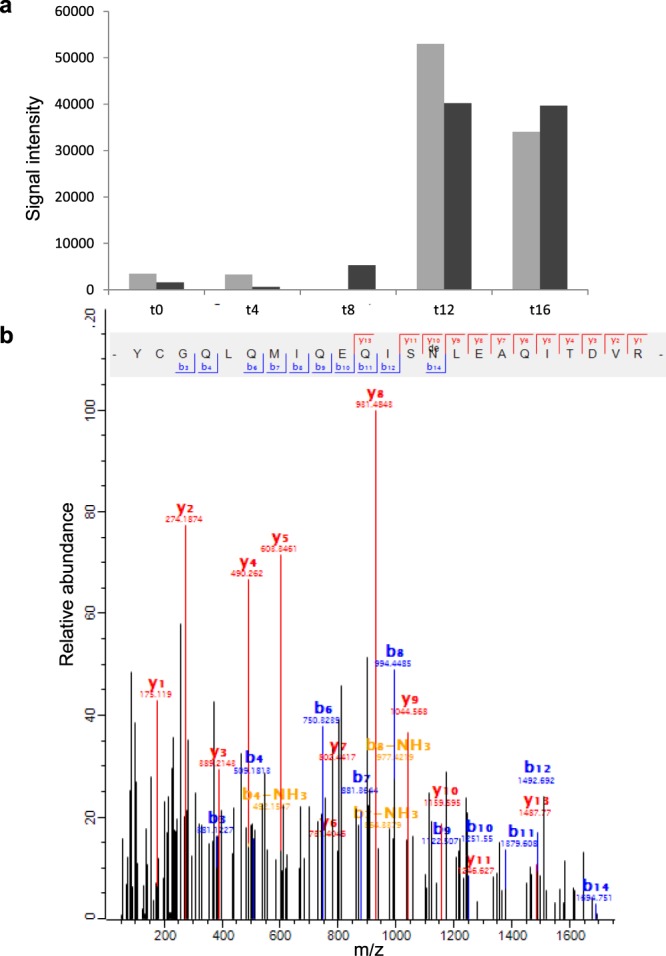
Example of modified keratin peptide in ageing fingermarks. (**a**) Mass feature intensity changes over time for unmodified (grey bars) and modified (black bars) peptide species originating from K1C9. Fingermark ages are indicated by t (0, 4, 8, 12 and 16) and are given in days on the x-axis. Summed mean intensities are on the y-axis. Modifications included: deamidation, oxidation and combinations thereof. (**b**) MS/MS spectrum of a modified peptide originating from K1C9 (sequence: YCGQLQMIQEQISNLEAQITDVR). Fragment ions are given by b_n_ (blue) and y_n_ (red and orange). Deamidated amino acid residue is indicated by ‘de’ in the peptide sequence.

From Fig. 3, it can be seen that overall modification processes for K2C1, K22E, K1C9, K1C10, and DCD peak at 12 days of ageing and become slightly less eminent after this time period. In light of the temporal behaviour of the above potential age markers, protein modifications in fingermark residue thus seems to be linked to differential protein response. Such parallel changes of protein modification and abundance in fingermark residue are exemplified in Fig. 4.

### Donor contact study

Marks from fingers that had been contaminated with saliva, urine or vaginal fluid were also processed and analysed. Positive identifications for the saliva, urine and vaginal fluid contaminated fingers (n = 6 samples each) are listed in Supplementary Tables S3, S4 and S5. For saliva contaminated fingers, the presence of about 53% of the here obtained fingermark proteome could be confirmed by our data, whilst for the other body fluids this was slightly lower; urine: 29% and vaginal fluid: 44%. Importantly, the potential age-marker keratins could all be positively identified in these samples. DCD could only be identified in fingers contaminated with saliva. For each sample set, some potentially unique proteins for the respective bodily fluids could be observed. These included for saliva: Mucin-5B (MUC5B), Alpha-amylase-1 (AMY1), Salivary acidic proline-rich phosphoprotein 1 (PRPC), Carbonic anhydrase 6 (CAH6) and Cystatin-D (CYTD), and for urine: Uromodulin (UROM). For vaginal fluid, no unique proteins were observed. The remaining proteins were found not to be specific for the here tested contaminants.

## Discussion

In this work, we combined an easily applicable sampling and protein extraction method, and high-end LC-MS analysis to obtain a first draft proteome map of fingermark residue. By means of efficient protein recovery using N_2_ drying instead of protein precipitation, as well as low-volume tryptic digestion we attempted straightforward sample preparation with minimal protein loss. The protein levels obtained from fingermarks (up to 57 μg) were however much lower compared to a previous study (128–542 μg)^16^. This importantly reveals the effects of ‘grooming’ prior to fingermark deposition, a procedure which was not included in our study so as to better mimic real-world conditions. In addition, Drapel *et al*.^16^ measured protein levels directly from the skin surface of fingertips rather than the much more realistic sampling from substrate applied in this work. It is nonetheless evident that the relatively low protein levels in fingermark residue make the application of MS-based protein sequencing challenging, notably in terms of stringent validation constraints. By using effective swab-sampling, low protein loss sample processing, and utilizing two-pass and match-between-runs database search strategies^29^ we were however able to overcome the limitations dictated by the protein yields from individual fingermarks. As was anticipated and confirmed by our data, our experimental design with maximum validity for crime investigation is likely to reveal high molecular variation between fingermarks. The here recorded data shows that proteome variation was not affected by donor sex nor by finger type. While this is in agreement with previous findings with regards to amino acids and lipids in fingermark residue^7^, donor sex differentiation has been successfully applied using matrix-assisted-laser-desorption/ionization MS profiling of fingermarks^9^. However, the precise molecular features that accounted for donor sex variation remained unclear from this study. Despite low and variable protein levels, we were able to explore the fingermark proteome and record temporal trends. This suggests that the proteins identified here are stable constituents of fingermark residue in view of e.g. donor traits and activities; an observation that could also be confirmed by the mere presence of common skin and sweat proteins.

According to GO analysis of the fingermark proteome described in this study, most positively identified species could be assigned to extracellular and intermediate filament proteins, which are critical for cell scaffolding, skin development and homeostasis, as well as to a group of keratins which are involved in epidermis development and terminal differentiation of keratinocytes. Keratins may originate from viable and non-viable cells, as previous microscopic and biomolecular studies have revealed that both nucleated keratinocytes and anucleated corneocytes occur in fingermark residue. The latter are however generally observed in higher abundance^30,31^ and regarded as death cells, notwithstanding the fact that they remain biologically active, being filled with keratin proteins and lipids^32,33^. The co-expression of many skin keratins, and CASPE, FLA, FLA2, DESP and Calmodulin-like protein 5 (CALL5) also suggests on-going (bio)chemical activity with regards to keratinocyte differentiation and physiology in fingermark residue after deposition^34–39^. Many of the less represented proteins in the here examined fingermarks could be associated with skin defence against pathogenic bacteria. Notably, PIP in excreted sweat has been reported to regulate non-immune defence^40^ probably by binding to IgG, CD4 and/or T cell receptors^41^. ZA2G has been shown to complex with PIP^41^ and although its exact function is yet unknown, the co-expression of these proteins suggests that defence mechanisms against bacteria are active in fingermark residue. As ZA2G also binds fatty acids and stimulates lipolysis^42,43^, this complex may also be involved in the degradation of fingermark residue. Other antibacterial proteins identified in this work include: S10A7, S10A8 and S10A9. Specifically, S10A7 is targeted against E.coli and has been previously associated with skin defence^44^, whilst S10A8 and 9 are commonly involved in the regulation of inflammatory processes and immune response^45^. Moreover, additional anti-inflammatory properties for *in vitro* S-nitrosylated S10A8 have been suggested^46^. Since the functioning of S100 family proteins in common keratinocyte proliferation, also in close relation with fatty acid processing, has a large evidence base as well, dual functionality of this protein family is likely. Other protein clusters in our samples highlight ALBU and HBB, probably acting as carrier proteins for keratins and S100 proteins. Albumin has also been recognized to contain regions with antioxidant functionality^47^. The remaining proteins identified here were not or only putatively associated with other fingermark residue constituents through protein-protein interaction analysis. One of these proteins is DCD, which is known for its involvement in skin defence, due to its antibacterial, antifungal and redox properties^48^. The latter are also expressed by THIO^49,50^. Ubiquitin peptide 52 (RL40) shows involvement of the proteasome system during keratinocyte proliferation^51^, whilst this is also confirmed by the co-expression of keratin 6b (K2C6B)^52^. Functioning of other members of the here reported fingermark proteome in regards to the biochemistry of skin surface material could not be inferred from the literature and, yet, remain elusive.

Temporal changes of protein abundance in fingermark residues were found for keratins 1, 2, 9, 10 and DCD. DCD exhibited a clear decrease in abundance with increasing age. Interestingly, most keratins showed, however, a relative increase of their peptide signals during the progress of ageing (after 8 days of ageing). Since our initial hypothesis was that prolonged exposure of fingermarks to the environment results in protein degradation and thus a decrease in protein signal, we assessed alternative scenarios supported by current literature. On the fingertip surface, there is a clear discrepancy in regards to recalcitrant molecular components (e.g. keratin proteins) and constituents that are generally more susceptible towards damage and degradation, such as small proteins, peptides and amino acids. It can thus be hypothesized that upon fingermark ageing the over-all sample matrix complexity decreases in parallel with a mass balance shift towards stable keratinaceous constituents. Therefore, it may be plausible that the observed time-dependent increase in keratin signals originate mostly from the relative better stability of this class of protein^53^.

Other processes which have to be taken under consideration when understanding the ageing trends recorded in this work are based on the assumption that (bio)chemically active skin cells are abundantly present in fingermark residue, as reported by Moret *et al*.^54^. In skin, enhanced expression of (keratin) proteins has been reported to be linked to e.g. ageing, UV radiation, irritants, and is suggested to result from stress and inflammatory responses of keratinocytes, apoptosis, transepidermal water loss or skin moister loss^17–19,55,56^. Such a response can be extrapolated to ageing fingermarks, especially because of the significant loss of water from fingermark residue in time^4,57^. It has also been shown that water loss results in increased epidermal calcium (Ca) levels and, in turn, increased protein expression^58^. As described earlier^54^, the concentration of ions (including Ca) in fingermark residue or sweat can increase as a result of water loss, and as such the assumption that an effect thereof on keratin expression is reasonable. The identification of specific Ca-binding proteins (S100 proteins, Calmodulin-like proteins) in this study also supports such a hypothesis.

For our age-biomarker panel total numbers of modification events gradually increased during ageing, and therefore modification processes seem to be linked to the increased detection of the keratin peptides. Specific trends for K2C1, K22E, K1C9 and K1C10 are in line with the above, although somewhat affected by sequence coverage (see Supplementary Fig. S3). Age-related increase of modifications in fingermarks may be expected from gradual exposure to the environment, in particular to moister and highly reactive species, such as oxygen (ROS)^59^. In many biological systems extensive modification may induce protein degradation and decreased protein turnover^59,60^, whilst this was not observed for these potential age markers identified. Our data suggest that, relatively low albeit increasing protein modification levels were achieved between 0 and 12 days of ageing. Reversible oxidation of methionine residues of proteins and thereby effectively consuming ROS^61^ may have contributed to protein accumulation during the ageing of fingermarks. Although more in-depth analysis of modification events of age-related markers would be very useful to extend these results, it is beyond the resolution of our data and the scope of this study. For these reasons, we did not further investigate protein modification in detail. Lastly, as fingermark residue was deposited on a foreign surface, changing biophysical conditions (e.g. temperature, pH, humidity) over time may also have contributed to enhanced cell permeability or rupture^32,62^, thereby releasing keratins to the environment or making them more susceptible for extraction. In view of the above discussion, the behaviour of DCD was as expected and showed a temporal decrease of LFQ levels, thereby suggesting peptide degradation or a more or less gradual consumption of this peptide in microbial defence. Modification events for this small protein follow this trend on the peptide level (see Supplementary Fig. S3).

The proteomes obtained from fingers contaminated with saliva, urine and vaginal fluid included all of the above age markers. Moreover, about 30–50% of the here reported fingermark proteome could be retrieved from these samples. This was to our surprise as we expected the significantly higher protein abundances in the tested bodily fluids to supress signals from endogenous fingermark proteins. In parallel, we were able to identify some highly contaminant-specific proteins in these samples, among many non-specific ones. Thus, given that potential age markers can be identified even after donor contact with (these) contaminants and the presence of proteins enabling contaminant sourcing, this additional study could be an important basis for further development of analytical protocols and data processing methods for age-biomarker discovery and validation studies. In prospect of such endeavours, it is noteworthy that keratin proteins are well-known to occupy various bodily fluids, including those studied here. It is, however, unclear how well such exogenous species are actually expressed in fingermark proteomes in view of the limited amount of proteinaceous material generally adhered to fingers.

For fingermark residue analysis in forensic contexts, environmental contamination is a critical and difficult issue that analytical protocols should address. Two approaches were taken here; (i) searching MS records against a contaminant database and (ii) assessment of a background proteome by using procedural blanks. As for (i), the contaminant database included all common contaminant proteins reported by the common Repository of Advantitious Proteins with the exception of keratins. Besides porcine trypsin (TRYP), introduced during sample preparation, these searches resulted in the identification of a keratin protein likely originating from sheep wool (Keratin, type I cytoskeletal 15, K1C15) and was considered as coming from dust contamination. For the procedural blanks that were used to monitor background contamination (ii), only low protein coverage of the recorded fingermark proteome was observed, with the exception of two medium abundance proteins likely introduced through carry-over action. Further, a keratin protein likely originating from sheep wool (Keratin, type II microfibrillar, component 5, K2M3) and porcine trypsin were observed in the blanks. These were also regarded as coming from dust contamination and sample preparation. Taken together, it can be assumed that swab-sampling and/or the fingermark substrate can introduce contamination. Yet, the intermittent occurrence of mass spectra originating from these contaminants support the integrity of the data presented here. Although the level contamination was low in this study, the chances of protein contamination occurring during fingermark exposure outside a controlled laboratory environment are generally high and inevitable, especially after longer exposure times. Similar to the procedural blanks used here, assessment of environmental contamination should be straightforward through sampling blank substrate in close vicinity to the target fingermarks. Also, cleaning of swabs prior to sampling by means of washing with a solvent suitable for upstream processing seems to be worthwhile to avoid additional contamination from the sampling procedure itself.

Limitations of this study mainly concern the univariate ageing conditions. By using glass as substrate there is a potential bias to detect ageing responses associated with non-reactive materials only. Variations in climatic conditions were also not studied here and this may add to the bias between laboratory conditions and forensic practice. However, the recalcitrance of keratin proteins under various and sometimes extreme conditions presents great promise for future forensic implementation.

In all, we established a sensitive fingermark sampling and proteomics approach, and present a first insight into the fingermark proteome. Moreover, four keratin proteins and the anti-microbial peptide Dermcidin are introduced as high potential biomarkers of fingermark age. Our study demonstrates that regardless of protein trace quantities and the presence of surface contaminants, LC-MS based fingermark proteomics is capable of providing a much deeper insight into the fingermark proteome compared to previously published methods. This is a very important step towards addressing forensic issues, such as age determination and providing additional tools for donor profiling. However, further improvements in sensitivity will expand the repertoire of fingermark proteins and establish a more complete database of the fingermark proteome. In addition, high-throughput targeted LC-MS approaches using multiplexed identification of forensically relevant protein or peptide markers will offer the necessary speed^63^ for examining fingermarks. The present study paves the way for applying marker-specific and time-resolved proteomics to obtain evidential and tactical information for crime investigation.

## Methods

### Fingermark collection and sampling

Fingermarks for the ageing study were collected by firmly pressing the tip of each finger (all five digits) onto a glass slide for 5 seconds. The samples were stored on paper towel in open document trays and aged for 4, 8, 12 and 16 days at 20 °C and 61% relative humidity. Each ageing pool, including non-aged samples (t = 0 days) consisted of 10 fingermarks from both male (n = 5) and female (n = 5) donors, and were processed and analysed individually. Glass slides without fingermarks (n = 10; aged and non-aged) were processed as below and analysed as procedural blanks. For the donor contact study (using two male and three female donors), the tip of each finger was briefly (1 min) brought in contact with the bodily fluids by the action of either mouth (saliva, n = 6 samples) or vaginal (vaginal fluid, n = 6 samples) insertion. As for mimicking contamination with urine, fingertips (n = 6 samples) were briefly dipped in urine, previously collected in plastic tubes. Fingertips were allowed to dry for approximately 10 min and fingermarks were collected on glass slides as above. Oral approval of this study was obtained from the TU Delft Research Ethics Committee and informed consent was obtained from all donors. All methods were performed in accordance with the relevant guidelines and regulations of the TU Delft and the Netherlands Forensic Institute.

Sampling of the slides was performed using polyester swabs (CleanTips Polyester Alpha, Texwipe, NC, USA) moistened with 50 μl extraction fluid containing 50% acetonitrile (ACN) in water (v/v). After removing the swabhandles using scissors, swabheads were placed in protein low-binding Eppendorf tubes and 200 μl of extraction fluid was added to each tube. Fingermark proteins were extracted from the swabheads by pulsed vortexing (10 mins) followed by sonication (10 min). The swabheads were then centrifuged for 10 min (11,300 × g) using spinbaskets. For each sample, the liquid permeate was dried under a N_2_ flow. Prior to swab sampling, all swabs were washed with 50% ACN in water (v/v) and centrifuged for 1 min (11,300 × g) using spinbaskets. This was repeated twice and then the swabs were further dried at RT for 1 h.

### In-solution trypsin digestion of fingermark proteins

Dried fingermark proteins were dissolved in 25.5 μl ammonium bicarbonate (50 mM) by pulsed vortexing (10 min) and sonication (10 min). Then the proteins were incubated at 95 °C for 5 min with 1.5 μl of DTT (100 mM). After cooling the samples to RT, 3 μl of IAA (100 mM) was added to each sample and incubated in the dark at RT for 20 min. Proteins were digested o/n at 37 °C with 0.5 μg trypsin. After digestion, the samples were either processed immediately or stored at −20 °C for a maximum of 24 h. The peptide solutions were purified and concentrated using solid-phase extraction (SPE). Here, C18 SPE spin-tips (Hypersep, SpinTip C18, Fisher Scientific, NH, USA) were conditioned twice with 50 μl of a solution containing 70% ACN, 29.9% water, 0.1% FA (v/v/v) and equilibrated three times with 50 μl 0.1% FA in water (v/v). After loading and binding of the peptides, the tips were washed three times with 50 μl 0.1% FA in water (v/v) and finally the peptides were eluted twice with 50 μl of a solution containing 70% ACN, 29.9% water and 0.1% FA (v/v/v). Reagents and samples were slowly forced through the spin-tips using air pressure. The eluted peptides were dried under a N_2_ flow and either processed immediately or stored at −20 °C for a maximum of 24 h.

### RP-ESI-MS/MS of proteolytic fingermark peptides

All samples were analysed by reversed-phase C18 liquid chromatography tandem MS (LC-MS/MS), using an Agilent HPLC system (1290 Infinity II, Agilent Technologies, CA, USA) connected to a high-resolution Agilent 6530 series electrospray ionization quadrupole time-of-flight mass spectrometer (ESI-QTOF-MS). Dried peptides were dissolved in 25 μl 0.1% FA in water (v/v) and 20 μl of each sample was loaded and separated on an Agilent AdvanceBio Peptide plus column (2.1 × 150 mm, 2.7 um C18 beads) at 40 °C. A binary solvent system was used consisting of mobile phases A (ACN containing 0.1% FA in water, v/v) and B (water containing 0.1% FA, v/v). Each run lasted 65 min and was composed of the following gradient: 5% A (isocratic, 0–5 min), 5–40% A (linear, 5–60 min), 40–95% A (linear, 60–63 min), 95% A (isocratic, 63–65 min). The flow rate was set to 0.3 ml/min. The mass spectrometer was operated at 2 GHz in extended dynamic range mode (low mass range; m/z 1700). Data dependent acquisition in positive ion mode was applied for MS analysis and was set to automatically select max. 3 precursor ions with intensities above 4500 counts for each cycle. Initial precursor selection was based on charge (order: +2, +3, >+3, unknown charge) followed by signal intensity. Peptide ions were fragmented by collision induced dissociation using collision energies based on charge state, m/z value and an offset value (+1 and +2: 3.1*m/z − 1, +3 and >+3: 3.6*m/z - 4.8). Full MS scans were acquired over a 200–1700 m/z range. Mass signals were selected for fragmentation at a scan-rate of 3 Hz, when exceeding a target count threshold of 25000 (counts/spectrum). Dynamic exclusion of precursor ions was set to 30 sec and static exclusion was used to reject mass signals from common non-peptidic fingermark contaminants, such as polyethylene glycols and detergents (see Supplementary Table S6 and Keller *et al*.^64^). Internal calibration was performed using two mass signals (m/z 121.12 and 922.01) from premixed standard solutions (HP-0921 and Purine) prepared according to manufacturer’s instructions.

### Peptide spectra database and modification searches

Raw files (Agilent.d files) were converted to peaklists using msconvert software (v3.0.11174)^65^, with vendor-based peak picking as only processing parameter. For the ageing study, a two-pass database search was performed using multiple search engines. First, error-tolerant searches were carried out using X!Tandem (Vengeance, v2015.12.15.2)^66^, MSGF + (v2017.01.13)^67^ and Andromeda (v1.5.6.0)^68^ algorithms through SearchGUI (v3.2.20) and Peptideshaker (v1.16.15) software^69^ using the following settings: proteolytic enzyme: trypsin, precursor mass tolerance: 20 ppm, fragment mass tolerance: 40 ppm, max. Missed cleavages: 2, charge range: 2–4, variable modification: Oxidation (M), fixed modification: Carbamidomethylation. All peaklists were searched against a target/decoy protein database containing all reviewed Uniprot human protein sequences (20,316 sequences, accessed March 2018) and 20,316 reversed decoy sequences. Search results were based on a complement of the posterior error probability (PEP)^70^ as described in Vaudel *et al*.^69^ and filtered at 25% FDR (false-discovery-rate) on both peptide and protein levels with a minimum number of peptide-spectrum-matches of ≥1 for each peptide and a minimum number of peptide matches ≥2 for each protein group. Based on the combined consensus results of these searches, a focussed database was constructed containing all sequences of the identified protein groups. This database (130 sequences) was appended with 19 sequences from common non- contaminant proteins (obtained from the common Repository of Advantitious Proteins; see http://www.thegpm.org/crap) and used for a second search (Andromeda engine only) combined with label-free quantification (LFQ) using MaxQuant software (v. 1.6.0.16)^71^. MaxQuant searches were performed using raw data files and with default settings (see Supplementary File 1), importantly including: proteolytic enzyme: trypsin, max. missed cleavages: 2, variable modification: Oxidation (M) and Alkylation (N-term), fixed modification: Carbamidomethylation. Protein identifications were filtered at a FDR of ≤1% using a target-decoy database search. Relative protein abundances were determined by the LFQ tool in the MaxQuant toolbox^29^. LFQ parameters were set to: fast LFQ with a min ratio count of 1 and matching between runs, and all peptide matches were used for quantification. All other LFQ parameters were set to default (see Supporting Information). Mass signals from the procedural blanks were searched as above. Protein modifications were determined using multi-blind unrestrictive searches using the MODa software tool (v1.61)^72^ and databases containing K2C1, K22E, K1C9, K1C10, and DCD sequences only. Settings for MODa were: data format: mgf, MS/MS instrument: ESI-QTOF, Target-Decoy: search with decoy proteins, AutoPMCorrection = 1, Fragment Tol.: 0.1 Da, Modified mass range: −150 to +400 Da, BlindMode = 2, HighResolution = ON, Enzyme: Trypsin. For the donor contact study, MS/MS recordings were interrogated using a 2-pass search; first the records were searched against the focussed fingermark database and then all peptide-spectrum-matches that remained unassigned were extracted and used for a second search against the above Uniprot human protein database. All searches for this study were performed using the Andromeda engine and processed using SearchGUI and peptideshaker software with identical parameter settings as described above apart from the FDR filters, which were set to ≤1%. In general, only proteins represented by two or more peptides occurring in at least two samples were regarded as positively identified. All MS data and MaxQuant results have been submitted to the proteomics identifications database (PRIDE) via ProteomeXchange, with dataset identifier PXD009706.

## Statistical Analyses

Effect sizes of ageing, donor sex and finger type were assessed using analysis of covariance (ANCOVA) and Bonferroni adjusted pairwise t-tests in R software^73^ (v. 3.3.3). A value of p < 0.05 was considered significant. Normality of our data was tested using quantile-quantile plots.

Protein functional networks and GO enrichments were obtained through the STRING database and accompanying software (https://string-db.org).

## Electronic supplementary material

Supplementary information

TableS3

TableS4

TableS5

TableS6

TableS7

MaxQuant settings
