# Supplementary material for: Proteomics as a new tool to study fingermark ageing in forensics
Source: Sci Rep. 2018 Nov 6;8:16425. doi: 10.1038/s41598-018-34791-z (PMC6219553; doi:10.1038/s41598-018-34791-z)
Supplement: Supplementary file 1 — Supplementary information [file 41598_2018_34791_MOESM1_ESM.docx]

**Supplementary Information**

**Proteomics as a new tool to study fingermark ageing in forensics**

Stijn Oonk^1,2^, Tom Schuurmans^1^, Martin Pabst^3^, Louis C.P.M de Smet^2,4^, Marcel de Puit^1,2^

^1^ Netherlands Forensic Institute, Digital Technology and Biometrics, Laan van Ypenburg 6, 2497 GB, Den Haag, Netherlands

^2^ Delft University of Technology, Faculty of Applied Sciences, Department of Chemical Engineering, Van der Maasweg 9, 2629 HZ, Delft, The Netherlands

^3^ Delft University of Technology, Faculty of Applied Sciences, Department of Biotechnology, Van der Maasweg 9, 2629 HZ, Delft, The Netherlands

^4^ Wageningen University & Research, Laboratory of Organic Chemistry, Stippeneng 4, 6708 WE Wageningen, The Netherlands

**Total protein levels in single fingermarks**

Prior to the fingermark ageing study, total protein levels were assessed for 200 single fingermarks on plastic and aluminum substrates using ammonium bicarbonate (50 mM in water), phosphate buffered saline (10mM phosphate, 150 mM sodium chloride in water) and ethanol (40% and 60% in water, v/v) as protein extractants. Sampling and processing steps were as described in the methods section apart from the trypsin digestion steps, which were not performed here. For each sample, dried protein extracts were resuspended in 25 μl ammonium bicarbonate (50mM) and analyzed using a bicinchoninic acid (BCA) assay (Pierce BCA protein assay kit, obtained from ThermoFisher) according to manufacturer’s instructions. After subtraction of mean background protein levels (n=40 blank substrates), protein levels in single fingermarks were found to range from 0.2 – 57.0 μg. Another 30 fingermarks on glass substrates were sampled and extracted with Acetonitrile (50% in water, v/v; identical to the ageing study). These samples were also processes as above and analysed by NanoDrop spectrophotometry (NanoDrop 1000, ThermoFisher) according to manufacturer’s instructions using direct absorbance at 280 nm. Protein levels for these samples ranged from 0.15 to 45.0 μg after background subtraction (n=5). In all, total protein levels in single fingermarks were inferred to range from about 0.2 to 51.0 μg.

**Distribution of identified proteins over the sample pools**


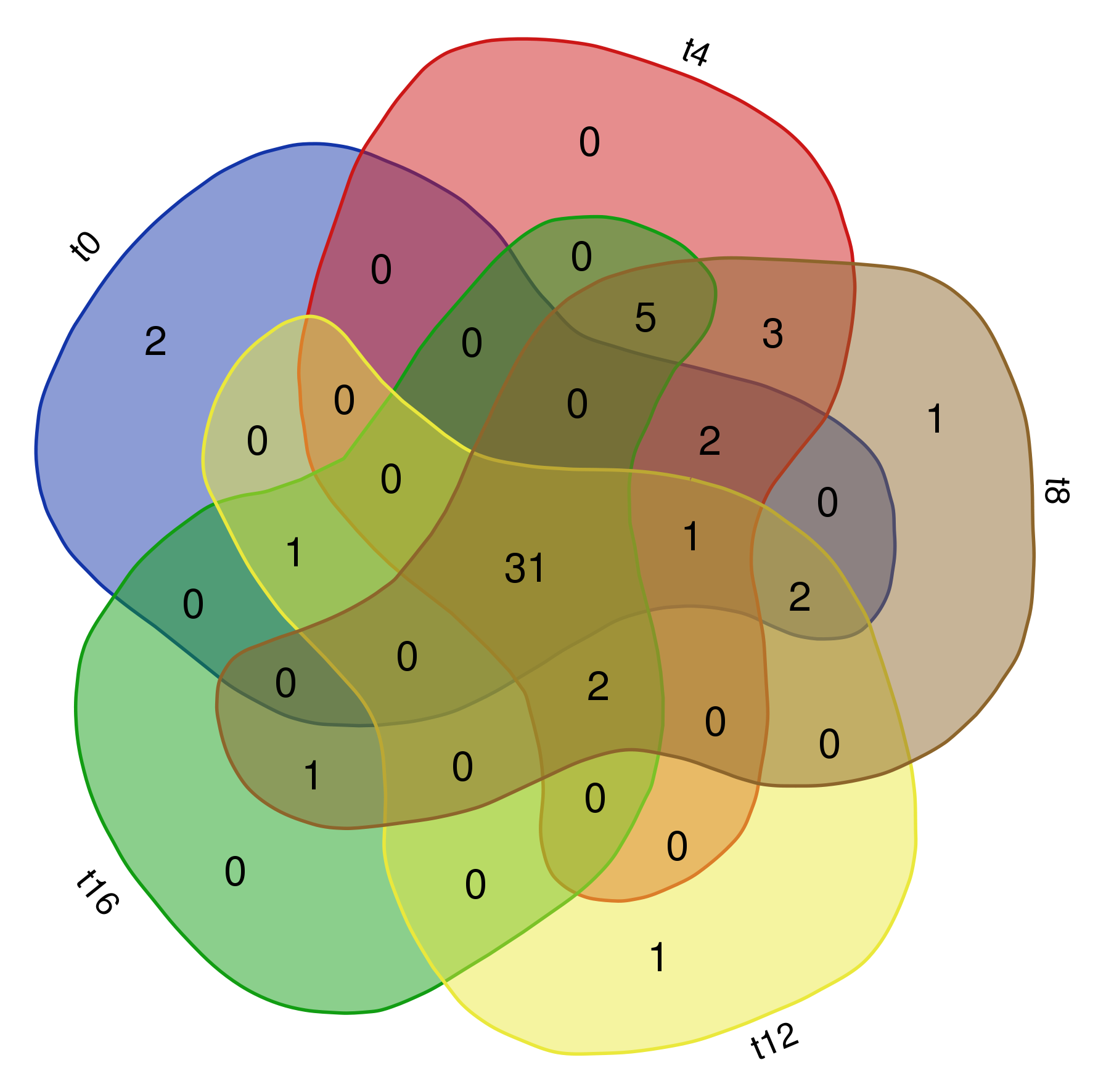


Figure S1. Venn diagram showing the distribution of the obtained fingermark proteome over the tested ageing pools. t(0, 4, 8, 12 and 16) indicates the age of the fingermarks in days.

**Gene Ontology (GO) analysis**

a


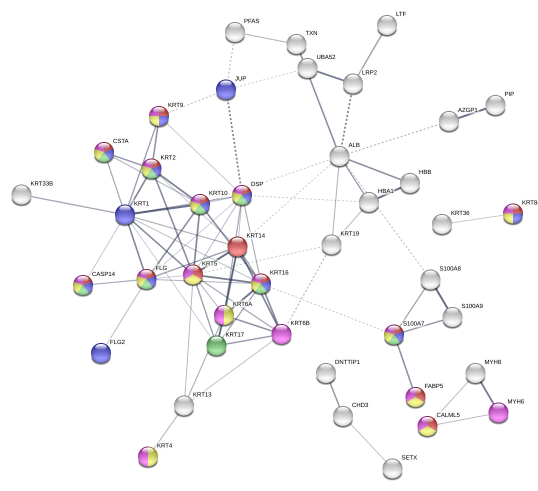


b


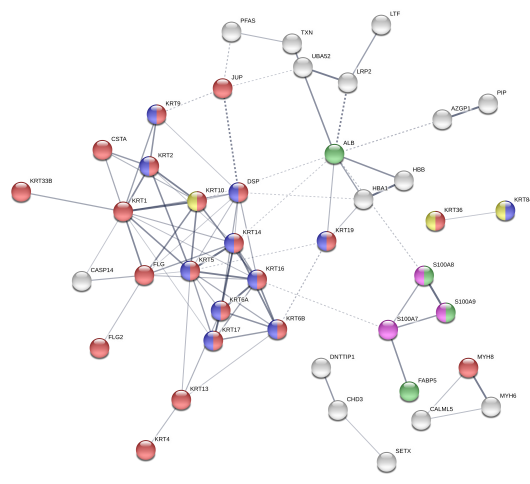


c


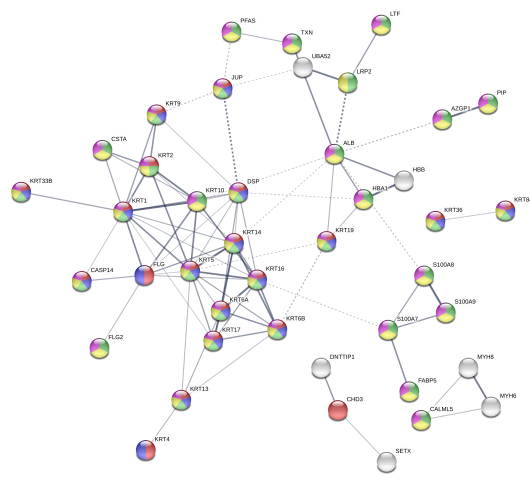


Figure S2. STRING network, Markov clusters and GO annotations of the identified fingermark proteins. The proteins (nodes) are represented by their gene names and functional associations are indicated by the gray lines. Their confidence level is given by the thickness of the lines, ranging from 0.150 (dotted lines) to 0.900 (dark gray lines). Top 5 GO annotations (based on false-discovery-rates) are given for biological processes (a), molecular functions (b) cellular components (c), and are represented by the color of the nodes; a: red (epidermis development), yellow (epithelium development), green (keratinocyte differentiation), blue (skin development), purple (tissue development), b: red (structural molecule activity), yellow (structural constituents of epidermis), green (fatty acid binding), blue (structural constituents of cytoskeleton), purple (RAGE receptor binding), c: red (intermediate filament cytoskeleton), yellow (extracellular region part), green (extracellular exosome), blue (intermediate filament), purple (membrane-bound vesicle).

Table S1: Analysis of covariance (ANCOVA) for potential age markers

|  |  |  |  |  |
| --- | --- | --- | --- | --- |
|  |  |  |  |  |
|  |  |  |  |  |
| **K2C1** | Df | F value | P value | Significance level |
| Age | 4 | 10.034686563 | **1.463138e-05** | 0.001 |
| sex | 1 | 0.001259556 | 9.718849e-01 |  |
| finger | 4 | 2.208922125 | 8.749083e-02 |  |
| sex:finger | 4 | 1.700288926 | 1.713398e-01 |  |
| Residuals | 36 | NA | NA |  |
|  |  |  |  |  |
| **K1C10** |  |  |  |  |
| Age | 4 | 2.7082858 | **0.04533586** | 0.05 |
| sex | 1 | 0.3833802 | 0.53969866 |  |
| finger | 4 | 0.2557485 | 0.90424195 |  |
| sex:finger | 4 | 0.5421825 | 0.70574669 |  |
| Residuals | 36 | NA | NA |  |
|  |  |  |  |  |
| **K1C9** |  |  |  |  |
| Age | 4 | 3.56629474 | **0.01501621** | 0.05 |
| sex | 1 | 0.04273899 | 0.83738238 |  |
| finger | 4 | 0.74947405 | 0.56488346 |  |
| sex:finger | 4 | 0.36623218 | 0.83103891 |  |
| Residuals | 36 | NA | NA |  |
|  |  |  |  |  |
| **K22E** |  |  |  |  |
| Age | 4 | 4.3408783 | **0.005743433** | 0.01 |
| sex | 1 | 0.7203651 | 0.401632778 |  |
| finger | 4 | 0.1820936 | 0.946199886 |  |
| sex:finger | 4 | 0.2387516 | 0.914559555 |  |
| Residuals | 36 | NA | NA |  |
|  |  |  |  |  |
| **DCD** |  |  |  |  |
| Age | 4 | 2.573286 | **0.05411407** | 0.05 |
| sex | 1 | 2.149649 | 0.15128369 |  |
| finger | 4 | 2.263423 | 0.08140913 |  |
| sex:finger | 4 | 1.463405 | 0.2336042 |  |
| Residuals | 36 | NA | NA |  |

Table S2. Abundance (LFQ) differences in time for potential age markers.

| **K2C1** |  |  |  |  |
| --- | --- | --- | --- | --- |
| Age | t0 | t4 | t8 | t12 |
| t4 | 1 | - | - | - |
| t8 | 1 | 1 | - | - |
| t12 | **0.00358** | **0.04071** | **0.02993** | - |
| t16 | **0.01766** | **0.04891** | **0.00179** | 1 |
|  |  |  |  |  |
| **K22E** |  |  |  |  |
| Age | t0 | t4 | t8 | t12 |
| t4 | 1 | - | - | - |
| t8 | 1 | 1 | - | - |
| t12 | **0.0352** | 0.0603 | 1 | - |
| t16 | **0.0396** | 0.4258 | 1 | 1 |
|  |  |  |  |  |
| **K1C9** |  |  |  |  |
| Age | t0 | t4 | t8 | t12 |
| t4 | 1 | - | - | - |
| t8 | 1 | 1 | - | - |
| t12 | **0.00435** | 0.68003 | **0.00870** | - |
| t16 | 0.34158 | 1 | 0.54066 | 1 |
|  |  |  |  |  |
| **K1C10** |  |  |  |  |
| Age | t0 | t4 | t8 | t12 |
| t4 | 1 | - | 1 | - |
| t8 | 1 | 1 | 1 | - |
| t12 | 1 | 0.3002 | **0.0165** | - |
| t16 | 1 | 1 | 0.0696 | 0.2068 |
|  |  |  |  |  |
| **DCD** |  |  |  |  |
| Age | t0 | t4 | t8 | t12 |
| t4 | 1 | - | - | - |
| t8 | 0.837 | 1 | - | - |
| t12 | 1 | 0.424 | 1 | - |
| t16 | 1 | 1 | 1 | 1 |

*Protein names are given underlined and in bold. Fingermark ages are indicated by t(0, 4, 8, 12, 16) and are given in days. Numbers represent p-values as obtained from Bonferroni corrected pairwise t-test; significant differences (significance level: 0.05) are given in bold.*

**Age-biomarker modifications in fresh and aged fingermarks**

*
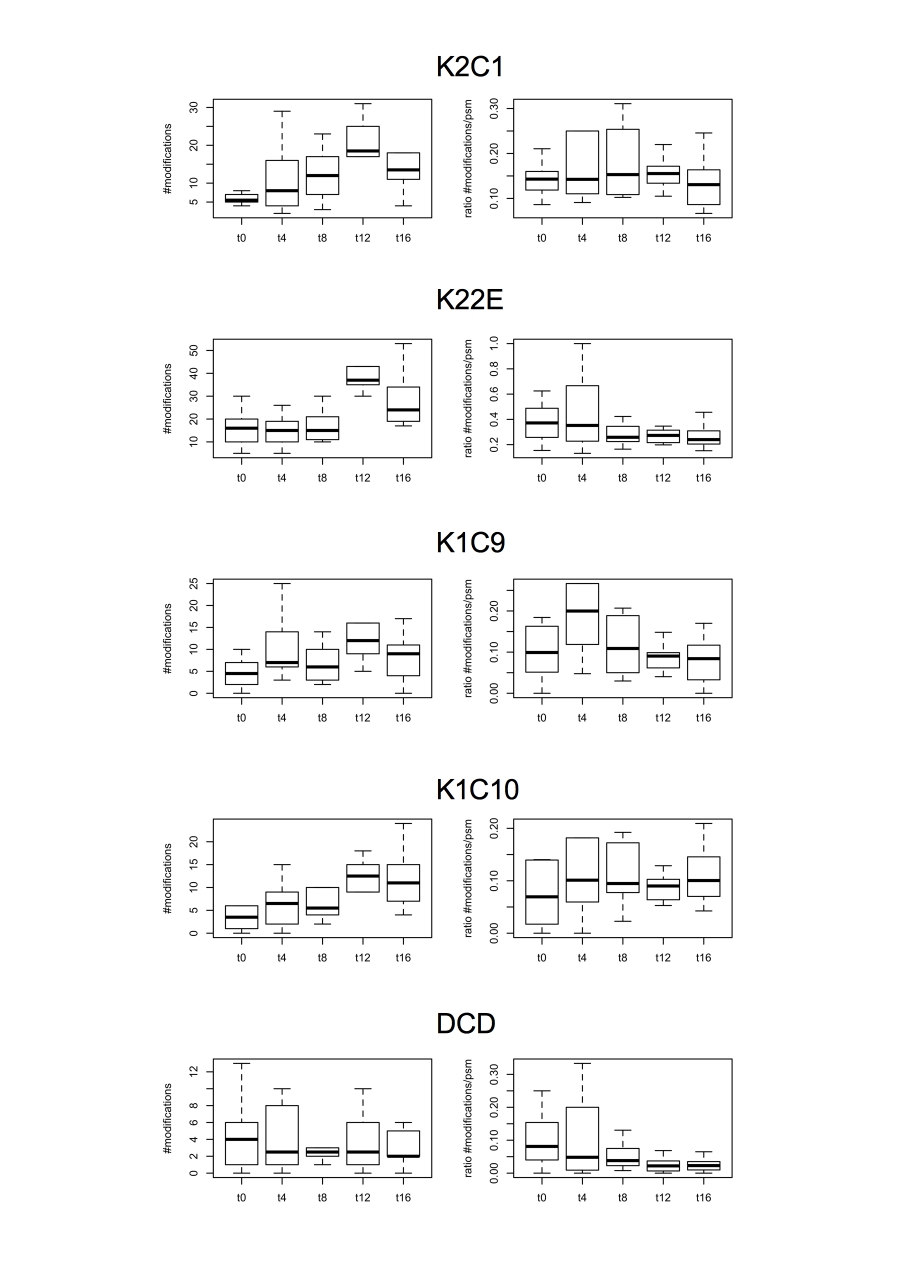
*

Figure S3. Protein-specific distribution of the observed modifications for potential ageing markers K2C1, K22E, K1C9, K1C10 and DCD over time, measured as total numbers of mass shifts (left panel) and fraction of total peptide-spectrum-matches (psm) (right panel). Fingermark ages are indicated by t (0, 4, 8, 12 and 16) and are given in days on the x-axis. Note that usual sample preparation and MS analysis induced mass shifts or adducts are not taken into account here.
