# Supplementary material for: Proteomics as a new tool to study fingermark ageing in forensics
Source: Sci Rep. 2018 Nov 6;8:16425. doi: 10.1038/s41598-018-34791-z (PMC6219553; doi:10.1038/s41598-018-34791-z)
Supplement: Supplementary file 7 — MaxQuant settings [file 41598_2018_34791_MOESM7_ESM.docx]

<?xml version="1.0" encoding="utf-8"?>

<MaxQuantParams xmlns:xsd="http://www.w3.org/2001/XMLSchema" xmlns:xsi="http://www.w3.org/2001/XMLSchema-instance" aifSilWeight="0" aifIsoWeight="0" aifTopx="0" aifCorrelation="0" aifCorrelationFirstPass="0" aifMinMass="0" aifMsmsTol="0" aifSecondPass="false" aifIterative="false" aifThresholdFdr="0" writeMsScansTable="true" writeMsmsScansTable="true" writePasefMsmsScansTable="true" writeAccumulatedPasefMsmsScansTable="true" writeMs3ScansTable="true" writeAllPeptidesTable="true" writeMzRangeTable="true" writeMzTab="false" disableMd5="false" connected="false" cacheBinInds="true" etdIncludeB="false" complementaryTmtCollapseNplets="true" connectedScore0="0.8" connectedScore1="0.9" connectedScore2="1">

<name>session1</name>

<maxQuantVersion>1.6.0.16</maxQuantVersion>

<tempFolder />

<numThreads>8</numThreads>

<sendEmail>false</sendEmail>

<fixedCombinedFolder />

<ionCountIntensities>false</ionCountIntensities>

<verboseColumnHeaders>false</verboseColumnHeaders>

<fullMinMz>-1.7976931348623157E+308</fullMinMz>

<fullMaxMz>1.7976931348623157E+308</fullMaxMz>

<calcPeakProperties>false</calcPeakProperties>

<showCentroidMassDifferences>false</showCentroidMassDifferences>

<showIsotopeMassDifferences>false</showIsotopeMassDifferences>

<filePaths />

<experiments />

<fractions />

<ptms />

<paramGroupIndices />

<parameterGroups />

<fixedModifications>

<string>Carbamidomethyl (C)</string>

</fixedModifications>

<fastaFiles>

<string>/string>

</fastaFiles>

<fastaFilesProteogenomics />

<fastaFilesFirstSearch />

<fixedSearchFolder />

<andromedaCacheSize>350000</andromedaCacheSize>

<advancedRatios>true</advancedRatios>

<pvalThres>0.005</pvalThres>

<neucodeRatioBasedQuantification>false</neucodeRatioBasedQuantification>

<neucodeStabilizeLargeRatios>false</neucodeStabilizeLargeRatios>

<rtShift>false</rtShift>

<separateLfq>false</separateLfq>

<lfqStabilizeLargeRatios>true</lfqStabilizeLargeRatios>

<lfqRequireMsms>true</lfqRequireMsms>

<decoyMode>revert</decoyMode>

<boxCarMode>all</boxCarMode>

<includeContaminants>false</includeContaminants>

<maxPeptideMass>4600</maxPeptideMass>

<epsilonMutationScore>true</epsilonMutationScore>

<mutatedPeptidesSeparately>true</mutatedPeptidesSeparately>

<proteogenomicPeptidesSeparately>true</proteogenomicPeptidesSeparately>

<minDeltaScoreUnmodifiedPeptides>0</minDeltaScoreUnmodifiedPeptides>

<minDeltaScoreModifiedPeptides>6</minDeltaScoreModifiedPeptides>

<minScoreUnmodifiedPeptides>0</minScoreUnmodifiedPeptides>

<minScoreModifiedPeptides>40</minScoreModifiedPeptides>

<secondPeptide>true</secondPeptide>

<matchBetweenRuns>true</matchBetweenRuns>

<matchUnidentifiedFeatures>false</matchUnidentifiedFeatures>

<matchBetweenRunsFdr>false</matchBetweenRunsFdr>

<dependentPeptides>false</dependentPeptides>

<dependentPeptideFdr>0</dependentPeptideFdr>

<dependentPeptideMassBin>0</dependentPeptideMassBin>

<msmsConnection>false</msmsConnection>

<ibaq>false</ibaq>

<top3>false</top3>

<independentEnzymes>false</independentEnzymes>

<useDeltaScore>false</useDeltaScore>

<splitProteinGroupsByTaxonomy>false</splitProteinGroupsByTaxonomy>

<taxonomyLevel>Species</taxonomyLevel>

<avalon>false</avalon>

<nModColumns>3</nModColumns>

<ibaqLogFit>false</ibaqLogFit>

<razorProteinFdr>true</razorProteinFdr>

<deNovoSequencing>false</deNovoSequencing>

<deNovoVarMods>true</deNovoVarMods>

<massDifferenceSearch>false</massDifferenceSearch>

<minPepLen>7</minPepLen>

<peptideFdr>0.01</peptideFdr>

<proteinFdr>0.01</proteinFdr>

<siteFdr>0.01</siteFdr>

<lfqSitePeptide>false</lfqSitePeptide>

<minPeptideLengthForUnspecificSearch>8</minPeptideLengthForUnspecificSearch>

<maxPeptideLengthForUnspecificSearch>25</maxPeptideLengthForUnspecificSearch>

<useNormRatiosForOccupancy>true</useNormRatiosForOccupancy>

<minPeptides>1</minPeptides>

<minRazorPeptides>1</minRazorPeptides>

<minUniquePeptides>0</minUniquePeptides>

<useCounterparts>false</useCounterparts>

<advancedSiteIntensities>true</advancedSiteIntensities>

<customProteinQuantification>false</customProteinQuantification>

<customProteinQuantificationFile />

<minRatioCount>2</minRatioCount>

<restrictProteinQuantification>true</restrictProteinQuantification>

<restrictMods>

<string>Oxidation (M)</string>

<string>Acetyl (Protein N-term)</string>

</restrictMods>

<matchingTimeWindow>0.7</matchingTimeWindow>

<alignmentTimeWindow>20</alignmentTimeWindow>

<numberOfCandidatesMultiplexedMsms>25</numberOfCandidatesMultiplexedMsms>

<numberOfCandidatesMsms>15</numberOfCandidatesMsms>

<massDifferenceMods />

<mainSearchMaxCombinations>200</mainSearchMaxCombinations>

<stackPeaks>false</stackPeaks>

<ms2PrecursorShift>0</ms2PrecursorShift>

<complementaryIonPpm>20</complementaryIonPpm>

<variationParseRule />

<msmsParamsArray>

<msmsParams Name="FTMS" MatchToleranceInPpm="true" DeisotopeToleranceInPpm="true" DeNovoToleranceInPpm="true" Deisotope="true" Topx="12" TopxInterval="100" HigherCharges="true" IncludeWater="true" IncludeAmmonia="true" DependentLosses="true" Recalibration="false">

<MatchTolerance>20</MatchTolerance>

<DeisotopeTolerance>7</DeisotopeTolerance>

<DeNovoTolerance>10</DeNovoTolerance>

</msmsParams>

<msmsParams Name="ITMS" MatchToleranceInPpm="false" DeisotopeToleranceInPpm="false" DeNovoToleranceInPpm="false" Deisotope="false" Topx="8" TopxInterval="100" HigherCharges="true" IncludeWater="true" IncludeAmmonia="true" DependentLosses="true" Recalibration="false">

<MatchTolerance>0.5</MatchTolerance>

<DeisotopeTolerance>0.15</DeisotopeTolerance>

<DeNovoTolerance>0.25</DeNovoTolerance>

</msmsParams>

<msmsParams Name="TOF" MatchToleranceInPpm="true" DeisotopeToleranceInPpm="false" DeNovoToleranceInPpm="false" Deisotope="true" Topx="10" TopxInterval="100" HigherCharges="true" IncludeWater="true" IncludeAmmonia="true" DependentLosses="true" Recalibration="false">

<MatchTolerance>40</MatchTolerance>

<DeisotopeTolerance>0.01</DeisotopeTolerance>

<DeNovoTolerance>0.02</DeNovoTolerance>

</msmsParams>

<msmsParams Name="Unknown" MatchToleranceInPpm="false" DeisotopeToleranceInPpm="false" DeNovoToleranceInPpm="false" Deisotope="false" Topx="8" TopxInterval="100" HigherCharges="true" IncludeWater="true" IncludeAmmonia="true" DependentLosses="true" Recalibration="false">

<MatchTolerance>0.5</MatchTolerance>

<DeisotopeTolerance>0.15</DeisotopeTolerance>

<DeNovoTolerance>0.25</DeNovoTolerance>

</msmsParams>

</msmsParamsArray>

<compositionPrediction>0</compositionPrediction>

<quantMode>0</quantMode>

<variationMode>none</variationMode>

</MaxQuantParams>
